# Dyslipidemia After Kidney Transplantation and Correlation With Cyclosporine Level

**DOI:** 10.5812/numonthly.11890

**Published:** 2013-06-14

**Authors:** Mahboobeh-Sadat Hosseini, Zohreh Rostami, Behzad Einollahi

**Affiliations:** 1 Nephrology and Urology Research Center, Baqiyatallah University of Medical Sciences, Tehran, IR Iran

**Keywords:** Kidney Transplantation, Dyslipidemias, Cyclosporine

## Abstract

**Background:**

Dyslipidemia after kidney transplantation is a frequent finding and is multifactorial. Immunosuppressive agents such as cyclosporine (CsA) can cause hypercholesterolemia.

**Objectives:**

As there were few reports with conflicting evidence on whether CsA related dyslipidemia is dose related and that CsA monitoring assays (trough level, C0, or two hour post dose level, C2) is a better predictor for dyslipidemia development; hence, the current study, in a large sample size, was designed to answer these questions.

**3. Patients and Methods:**

In the current retrospective cross sectional study, 1391 kidney transplant recipients were enrolled. All patients received CsA plus mycophenolatemofetile or azathioprine and prednisolone. Serum creatinine, CsA blood levels and lipid profile were measured after 12-14 h fasting. Mann-Whitney and Kruskal-Wallis, Pearson`s test and logistic regression were used for data analyses.

**Results:**

Mean age of 1391studied population was 38.7 ± 15 years old. Hypercholesterolemia and hypertriglyceridemia were observed in 58.9% and 86.6%, respectively and they were more significantly detected in cadaveric kidney transplantation. Dyslipidemia had weak correlation with age of recipient, serum creatinine, C0 and C2 levels of CsA. At logistic regression, serum creatinine was the only risk factor for hypercholesterolemia development after kidney transplantation (OR = 1.6, CI 95%: 1.4 -1.8).

**Conclusions:**

Dyslipidemia is a common finding after kidney transplantation and has no correlation with CsA level. According to conflicting data on the precise effect of different factors in inducing dyslipidemia, prospective large sample size studies should consider better control of dyslipidemia.

## 1. Background

Lipid abnormalities are frequent findings among renal transplantation recipients, although renal function is often normal or near normal (1). Besides other contributing factors, post-transplant dyslipidemia may be related to immunosuppressive treatment (2, 3). Cyclosporine (CsA) can directly cause post-transplant hypercholesterolemia, an effect that is independent of concurrent (4). Also the impact of CsA upon lipid levels appears to be dose dependent. The trough blood (C0) levels of the agents correlate with elevations in total and LDL cholesterol concentrations and with reduction in HDL cholesterol levels corticosteroid use (3). Due to high incidence of atherosclerosis events in the renal transplantation population, it should be considered a coronary heart disease equivalent risk and ability to modify dyslipidemia render lipid modification which is a potentially important intervention for improving outcome after kidney transplantation (5-9).

## 2. Objectives

There are conflicting evidence and few reports on whether CsA-related side effects are dose dependent phenomenon and if C0 level is a better predictor than two hour post dose (C2) level of CsA for dyslipidemia evolution (5, 7-10). To answer these questions, the current study evaluated correlation among dyslipidemia, C0 and C2 levels of CsA in a large sample.

## 3. Patients and Methods

### 3.1. Study Population

In the current retrospective, cross-sectional study, 1391 kidney transplant recipients in several Kidney Transplant Centers, Tehran, Iran from April 2008 to January 2011 were enrolled. During this period, all patients were referred to a single laboratory for measurement of all biochemical parameters tests such as lipid profile. The patients with any infections, rejections, hypothyroid state or patients who used beta belockers, thiazids or OCPs were excluded. The current study was approved by the Local Ethics Committee of Baqiyatallah University of Medical Sciences.

### 3.2. Immunosuppression 

All patients received CsA (targeting a trough level of 200 to 300 ng/mL for the first 3 months, 100 to 250 ng/mL for 4 to 12 months and 100 to 150 ng/mL thereafter; while using target of 2 hour post dose level was 800 to 1000 ng/mL for the first 3 months and 400 to 600 ng/mL for subsequent months) plus mycophenolatemofetil (1–2 g per day) or azathioprine (1–2 mg/kg per day) and prednisolone (initially 1 mg/kg daily with tapering to 5-10 mg per day during 3 to 6 months).

### 3.3. Clinical and Biochemical Data Collection

The clinical and biochemical data recorded for all patients were sex of recipients and donors, donor source (living or cadaveric), serum creatinine (Cr) concentration, CsA levels, and lipid profile (triglyceride (TG), cholesterol (Chol), HDL- cholesterol and LDL- cholesterol). All the mentioned biochemical variables were measured < 3 months, 4-12 months and > 1 year after kidney transplantations. Blood samples were obtained in the morning after 12-14 hours fasting.

### 3.4. Definitions

According to NCEP ATP III, (11) hypercholesterolemia and hypertriglyceridemia were considered if serum cholesterol and triglyceride concentrations were more than 240 mg/dL and 150 mg/dL respectively for at least two consecutive tests performed. LDL- cholesterol and HDL- cholesterol levels more than 130 and less than 40 mg/d were considered as high LDL and low HDL, respectively.

### 3.5. Statistical Analysis

Continuous variables were expressed mean ± SD. Since none of the variables had normal distribution, non-parametric tests (Mann-Whitney and Kruskal Wallis) were used to determine differences between the two and several groups. Pearson’s test was used to evaluate correlation between dyslipidemia and CsA blood levels. Data were analyzed using computer software program SPSS version 17.0 at P < 0.05 as level of significance.

## 4. Results

Mean age of 1391 subjects of the study population was 38.7 ± 15 years old, 849 (61%) male and 542 (39%) female. Seventy-five percent of patients received transplant kidney of living unrelated patient and 7% of living related patient and 6% of cadaveric (Table 1). Hypercholesterolemia and hypertriglyceridemia were observed in 58.9% and 86.6% of patients, respectively. Prevalence of high LDL and low LDL was 33.4% and 61.4%, respectively. Dyslipidemia was more common in female than male, but only high LDL was statistically significant. Hypercholesterolemia and hypertriglyceridemia were more significantly detected in cadaveric (Table 2).

**Table 1.** Baseline Characteristics of Study Population

Variables	Female	Male	Total
**Age, y**	37 ± 15^a^	39.7 ± 14^a^	38.7 ± 15^a^
**Sex**	542	849	1391
**Cr ^b^, mg/** **dL**	1.5 ± 0.9^a^	1.7 ± 0.8^a^	1.6 ± 0.9^a^
**Total cholesterol, mg/dL**	193.6 ± 48^a^	175.8±40^a^	181 ± 43.7^a^
**Triglyceride, mg/dL**	196.6 ± 47.7^a^	183.2 ± 43.9^a^	188.2 ± 45.8^a^
**LDL ^b^, mg/dL**	105 ± 35^a^	100 ± 33^a^	102.5 ± 34^a^
**HDL ^b^ , mg/dL**	47.7 ± 15.3^a^	48.7	47.7 ± 15.3^a^
**Co level, ng/mL**	152 ± 94.6^a^	151.8 ± 94.8^a^	151.9 ± 94.7^a^
**C2 level, ng/mL**	527.5 ± 175^a^	509 ± 175^a^	516.6 ± 175.9^a^

^a^Mean ± SD

^b^Abbreviations: Cr, creatinine; LDL, low density lipoprotein; HDL, high density lipoprotein

^c^C0, Trough level of CsA

^d^C2, 2-hr post-dose CsA level

**Table 2.** Dyslipidemia Prevalence After Kidney Transplantation

Variables	Hypercholesterolemia No. (%)	P value	Hypertriglyceridemia No. (%)	P value	Low HDL No. (%)	P value	High LDL No. (%)	p value
**Sex**		0.8		0.2		0.4		0.01
Female	349 (63.8)		495 (91.3)		346 (63.8)		275 (50.7)	
Male	473 (55.7)		710 (83.6)		473 (55.7)		364 (42.8)	
**Type of transplantation**		0.02		0.1		0.03		0.06
LURP ^a^	597 (57.5)		898 (86.5)		329 (39.3)		473 (61.9)	
LRP ^a^	62 (62.6)		86 (86.8)		39 (39.3)		47 (58)	
**Cadaveric**	58 (69)		78 (92.8)		34 (40.4)		35 (50.7)	

^a^Abbreviations: LRP, living related patient; LURP, living unrelated patient

The cholesterol and triglyceride level 4-12 months after transplantation were significantly higher than their level in less than 3 months and more than 1 year after kidney transplantation. The lowest level of HDL and the highest level of LDL were detected more than 1 year after transplantation (Table 3). Dyslipidemia had weak correlation with age and sex of recipient, creatinine level, C0 and C2 levels of CsA (Table 4).

**Table 3.** Lipid Profile Level According to After Transplantation Time

After Transplantation Time	Cholesterol	P value	Triglyceride	P value	HDL ^a^	P value	LDL ^a^	P value
**< 3 month**	176 ± 50	0.000	186.6 ± 93	0.000	49.7 ± 14	0.000	83 ± 31	0.000
**4-12 month**	189.7 ± 47.5		196.8 ± 116		47 ± 15		101.7 ± 34	
**> 12 month**	181.7 ± 42		164.6 ± 93.9		45 ± 14		102 ± 34	

^a^Abbreviations: LDL, low density lipoprotein; HDL, high density lipoprotein

**Table 4.** Dyslipidemia Correlation With Age of Recipient, Creatinine, C0 And C2 Level of Cyclosporine ^a^

	Hypercholesterolemia	Hypertriglyceridemia	Low HDL	High LDL
**Age of recipient**	0.15	0.1	0.09	0.15
**C0** **^b^**	-0.003	0.073	0.007	-0.02
**C2** **^c^**	0.18	0.16	0.09	0.12
**Cr**	-0.02	0.27	0.10	-0.1

^a^Correlation coefficient has been used

^b^C0, Trough level of CsA

^c^C2, 2-hr post-dose CsA level

## 5. Discussion

Dyslipidemia is a common complication after renal transplantation and hypertriglyceridemia is the most common type of dyslipidemia, although the current study finding on dyslipidemia prevalence is compatible with other studies, according to different dyslipidemia definitions, the type of dyslipidemia is not similar in studies (1, 12-16).

Hypercholesterolemia and low HDL were significantly higher in cadaveric than living unrelated and related patients, probably due to more prevalent delayed or impaired graft function following cadaveric kidneys, which need more immunosuppressive drug than living kidney transplantation (17). Dyslipidemia is associated with cardiovascular complication and graft atherosclerosis (18). Therefore, lipid modification is an important intervention to improve outcomes. CsA and steroid are the major causes of lipid abnormalities. Hence, immunosuppressive therapy optimizing, can be a major priority in the management of kidney transplant recipients (10, 19).

In the present study, trough CsA concentration and C2 level of CsA were used to guide therapy. Precision of these parameters is related to strong correlation between them and dyslipidemia development. The current study found weak correlation between dyslipidemia, trough and C2 level of CsA. Despite the stable renal function in the patients under study, slight decrease in creatinine clearance can result in decreasing cholesterol clearance and hypercholesterolemia. The major power of the current study was the large sample size, and the most important limitations were not being prospectively designed, lack of data regarding diabetes, statins using, and BMI.

In the current study dyslipidemia was a common finding after kidney transplantation and had no correlation with CsA level but serum creatinine concentration and female sex are two major predictors for hypercholesterolemia and hypertriglyceridemia development respectively. Therefore, to reduce hypercholesterolemia after kidney transplantation normal renal function maintaining should be strongly considered.
